# Dr. White on Insanity

**Published:** 1914-11

**Authors:** 


### Dr. White on Insanity.

Recommendations.—Dr. F. S. White, Superintendent of the Southwestern Insane Asylum, in his annual report to the Governor, recommends, among other things, that the name "insane asylum" be changed to "State Hospital," inasmuch as these institutions, as now conducted, are for the treatment and cure of mental diseases, rather than as a mere place for confinement. He further recommends that the present law by which one is committed to the asylum be repealed, stigmatizing it as a monstrosity, unnecessarily expensive, extremely offensive and harmful in sensitive cases.

What would be of very great importance, the doctor suggests that an ample appropriation be made to enable efficient research work to be carried on in the study of the cause and pathology of the insane. It is by just such investigation as this, together with better registration of vital statistics, that we can acquire the fundamental information necessary to direct a correct understanding of the laws of eugenics.

*Increase of Insanity.*—Dr. White's report indicates that there are approximately 238 more cases now in the State than when he made his report two years ago. This is exclusive of the three asylums and represents those confined to jails, poor farms and that are still with relatives.

*Causes of Insanity.*—There is not an influence, says the doctor, either external or internal, pleasurable or painful, normal or abnormal, voluntary or involuntary, which does not react in some way upon the mind. People are differently endowed; what would affect one would not affect another; likewise, the exciting cause is very varied and may affect the most unexpected persons. Many of these influences are under control of the individual, but others are handed down to him by a pernicious ancestry and are beyond his control. Such is heredity, which is passed down from generation to generation, little affected by the life and environment of the victims.

"Men and women must be taught," says the doctor, "that it is inevitable that they will transmit to the offspring the weakness, frailties and unstableness which they themselves possess. People realize and act upon this potentiality in the breeding of stock, but forget it in the breeding of children, and the result is manifested in the great number of defectives which encumber the earth and burden the more fortunate and favored class."

The doctor very properly assigns to syphilis the very extensive part it plays in the rôle of insanity and nervous diseases. He says, "acquired in the dark, concealed from the light, its ravages are so insidious and its symptoms so protean that until within the last few years it was overlooked in many cases."

The hardships and monotony of farm life without sufficient diversion, the underfed condition, personal sacrifices and too frequent child-bearing are potent factors in the causation of insanity, there being thirty cases attributed to these conditions admitted during the last year.

"Diversion and change," says the doctor, "are as necessary to mental, moral and physical health in women as rain and sunshine are to the growth of vegetation. * * * Human nature is so constituted that amusement, diversion and change are absolutely necessary to health and happiness. He who can bring about an improvement in these conditions will be a great benefactor to a large part of the population of Texas."

The doctor urges the necessity of conforming to the rules and principles of right living as a check to the ever onward tendency towards a nervous instability which ultimately becomes an innate factor from which no man can escape.

In spite of the foregoing, the doctor very cheeringly says: "The human race is more perfect today than ever before. The human mind cannot conceive what it would have been had the people for the past ten generations properly appreciated and acted upon the effects of heredity. People have been made in a haphazard way. What would the race be today had people been made as we now make cattle, horses, hogs, etc.?

Surely the doctor's report is a strong plea for practical eugenics. Will we ever heed the warning?

---

Dr. M. M. Carrick, of Dallas, has been urged by many citizens and newspapers as the proper man for the position of State Health Officer under the new administration. If Governor Ferguson is sincere in his promise to give the State efficient men in the places he has to fill, he will appoint Dr. Carrick to the position and do the State a service as well as cast glory on himself.

---

THERE ARE 149 students registered in the Main University at Austin who are doing their first year of medical work, intending to continue their medical studies at the Medical Department at Galveston next year. They have formed what is known as the "Pre-Medico Society."

It is good news that despite war and the strenuous conditions of the country at this time the State medical college opened with a larger student body than last year.

With 235 students in Galveston, and 149 in Austin doing the first year's work in medicine, there need be no fear of a dearth of doctors in Texas.

---

A bequest of nearly $1,000,000 to the medical school of Western Reserve University has been announced recently by President Charles F. Thwing.

